# CIPR: a web-based R/shiny app and R package to annotate cell clusters in single cell RNA sequencing experiments

**DOI:** 10.1186/s12859-020-3538-2

**Published:** 2020-05-15

**Authors:** H. Atakan Ekiz, Christopher J. Conley, W. Zac Stephens, Ryan M. O’Connell

**Affiliations:** 1 Division of Microbiology and Immunology, Department of Pathology, University of Utah, 15 N. Medical Dr. East, JMRB, Salt Lake City, UT 84112 USA; 2 Huntsman Cancer Institute, University of Utah, 2000 Circle of Hope Dr, Salt Lake City, UT 84112 USA; 3 Bioinformatics Shared Resource, Hunstman Cancer Institute, University of Utah, 2000 Circle of Hope Dr, Salt Lake City, UT 84112 USA

**Keywords:** Single cell RNA-sequencing, Cluster analysis, Identity prediction, Similarity, Gene expression profiling, Immune cells

## Abstract

**Background:**

Single cell RNA sequencing (scRNAseq) has provided invaluable insights into cellular heterogeneity and functional states in health and disease. During the analysis of scRNAseq data, annotating the biological identity of cell clusters is an important step before downstream analyses and it remains technically challenging. The current solutions for annotating single cell clusters generally lack a graphical user interface, can be computationally intensive or have a limited scope. On the other hand, manually annotating single cell clusters by examining the expression of marker genes can be subjective and labor-intensive. To improve the quality and efficiency of annotating cell clusters in scRNAseq data, we present a web-based R/Shiny app and R package, *Cluster Identity PRedictor (CIPR)*, which provides a graphical user interface to quickly score gene expression profiles of unknown cell clusters against mouse or human references, or a custom dataset provided by the user. CIPR can be easily integrated into the current pipelines to facilitate scRNAseq data analysis.

**Results:**

CIPR employs multiple approaches for calculating the identity score at the cluster level and can accept inputs generated by popular scRNAseq analysis software. CIPR provides 2 mouse and 5 human reference datasets, and its pipeline allows inter-species comparisons and the ability to upload a custom reference dataset for specialized studies. The option to filter out lowly variable genes and to exclude irrelevant reference cell subsets from the analysis can improve the discriminatory power of CIPR suggesting that it can be tailored to different experimental contexts. Benchmarking CIPR against existing functionally similar software revealed that our algorithm is less computationally demanding, it performs significantly faster and provides accurate predictions for multiple cell clusters in a scRNAseq experiment involving tumor-infiltrating immune cells.

**Conclusions:**

CIPR facilitates scRNAseq data analysis by annotating unknown cell clusters in an objective and efficient manner. Platform independence owing to Shiny framework and the requirement for a minimal programming experience allows this software to be used by researchers from different backgrounds. CIPR can accurately predict the identity of a variety of cell clusters and can be used in various experimental contexts across a broad spectrum of research areas.

## Background

Single cell RNA sequencing (scRNAseq) has enabled researchers to interrogate cellular phenotypes at an unprecedented resolution and led to the discovery of several new biological phenomena [1]. This new tool has gained significant traction in numerous research areas including immunology, developmental and cancer biology, and is being continually improved in terms of the technology and analytical pipelines. Data from scRNAseq is typically analyzed via various unsupervised clustering approaches resulting in distinct groups of cells defined by similar gene expression profiles [2]. During scRNAseq data analysis, it is advantageous to relate single cell clusters to known cell types before proceeding to downstream steps such as differential expression analyses and data visualization. In the past 2 years, an increasing number of software solutions to automatically classify cell clusters in scRNAseq data have been reported [3–13]. Recent studies provided a rigorous assessment of these methods in terms of performance and accuracy, and found that no one tool is perfectly suitable for every experimental context [14–16]. These tools can be computationally intensive, and they generally lack a graphical user interface limiting their use in iterative analyses. Furthermore, these tools may not be easily adaptable to different experimental contexts and, in some cases, the learning curve can be difficult to those with limited coding experience due to specialized data structures. Thus, many researchers still rely on manually examining known marker genes to determine the cluster identity in scRNAseq experiments. However, manual annotation of cell clusters is labor-intensive and requires field-specific expert biological knowledge. Additionally, this approach can be subjective and difficult to reproduce. Therefore, there is a need for a fast and accurate classification algorithm that can be easily integrated into existing analytical pipelines.

To facilitate identifying cell clusters in scRNAseq data, here we present Cluster Identity PRedictor (CIPR) (pronounced cy-per), a web-based R/Shiny application and R package which scores complex multi-gene expression signatures of unknown experimental cell clusters against known reference cell populations. After calculating identity scores between unknown clusters-reference pairs, the pipeline generates informative graphical outputs allowing the users to easily assess predictions. CIPR pipeline can be used with one of the 7 preloaded mouse and human reference datasets, or with a user-provided custom reference dataset for specialized studies. CIPR algorithm performs internal gene name matching in a species-agnostic manner enabling comparisons across species. Users can exclude irrelevant reference cell subsets and lowly variable genes from the analysis and adapt the CIPR pipeline to their experimental needs. Our analyses using scRNAseq data obtained from mouse melanoma tumor-infiltrating immune cells show that CIPR can accurately and efficiently predict the identity of single cell clusters and can be used to annotate immune cell subsets. Benchmarking CIPR against 2 robust software solutions performing a similar task, SingleR [12] and scmap [13], revealed that CIPR produces comparable results while requiring significantly less compute resources and a shorter runtime. Thus, CIPR can facilitate scRNAseq data analysis by quickly and accurately annotating unknown cell clusters.

## Implementation

### Summary of the pipeline

Software is implemented using R programming language and Shiny framework. CIPR is accessible via online Shinyapps.io server [17], or as a stand-alone R package [18]. The open source code for the Shiny application and the R package is available on GitHub [18, 19]. CIPR can work with two types of experimental input data commonly generated by popular scRNAseq analysis software: **i)** a data frame containing differentially expressed genes per cluster and their log fold-change (logFC) values, **ii)** a summary data frame that contains average normalized log-counts per gene in each cluster (for all genes in the dataset). As the reference, CIPR allows users to select one of the 7 preloaded reference datasets **(**Table 1**)** or upload a custom reference dataset generated by various high throughput analysis approaches including microarray and RNAseq. Depending on the input type, CIPR can either compare the logFC values of differentially expressed genes in clusters against reference comparators (where a logFC value at individual gene level is calculated for each reference cell subset in comparison to the average expression across the entire reference dataset), or calculate correlations using the entire gene set. The algorithm computes a vector of identity scores through pairwise comparisons of clusters and reference samples, generates visualizations and allows downloading the results as a csv file. In the CIPR R package implementation, the pipeline functions as described above with added functionality to control the graphical and numerical output.

**Table 1:** Summary of the reference datasets included in CIPR

Reference dataset	Species	Number of samples/ features	Number of cell types (main/fine)	Reference cell types	Ref
Immunological Genome Project (ImmGen)	*M. musculus*	296/24197	20/296	B cell, Baso, DC, Eosino, gd-T, Gran, ILC-1, ILC-2, ILC-3, Mac, Mast, Mono, NK, NKT, Pre-B, Pre-T, Stem-Prog, Stromal, T cell, Treg	[20]
Presorted cell RNAseq (various tissues)	*M. musculus*	358/21214	18/28	Adipocyte, Astrocyte, B cell, Cardiomyocyte, DC, Endothelial, Epithelial, Erythrocyte, Fibroblast, Gran, Hepatocyte, Mac, Microglia, Mono, Neuron, NK, Oligodendrocyte, T cell	[21]
Blueprint/ENCODE	*H. sapiens*	259/19859	24/43	Adipocytes, B cell, T cell, Chondrocyte, DC, Endothelial, Eosino, Epithelial, Erythrocyte, Fibroblast, HSC, Keratinocyte, Mac, Melanocyte, Mesangial, Mono, Myocyte, Neuron, Neutro, NK cells, Pericyte, Skeletal muscle, Smooth muscle	[22, 23]
Human Primary Cell Atlas	*H. sapiens*	713/19363	37/157	Astrocyte, B cell, BM, Prog, Chondrocyte, CMP, DC, ESC, Endothelial, Epithelial, Erythroblast, Fibroblast, Gametocyte, GMP, Hepatocyte, HSC, iPS, Keratinocyte, Mac, MEP, Mono, MSC, Myelocyte, Neuroepithelial, Neuron, Neutro, NK, Osteoblast, Platelet, Pre/Pro-B, Smooth muscle, T cell, Tissue SC	[24]
Database of Immune Cell Expression (DICE)	*H. sapiens*	15*/57,773	5/15	CD4+ T cell, CD8+ T cell, NK cell, B cell, Mono	[25]
Hematopoietic differentiation	*H. sapiens*	211/13276	17/38	B cell, Baso, CD4+ T cell, CD8+ T cell, CMPs, DC, Eosino, Erythroid, GMP, Gran, HSC, Megakaryocyte, MEP, Mono, NK, NKT	[26]
Presorted cell RNAseq (PBMC)	*H. sapiens*	114/46077	11/29	B cells, Baso, CD4+ T cell, CD8+ T cell, DC, Mono, Neutro, NK cells, Prog, T cell	[27]

Table 1 Multiple reference datasets are available within the CIPR pipeline. These include 2 reference datasets from mouse and 5 reference datasets from human, which contain data from both immune and non-immune cells. ImmGen reference was prepared from raw microarray data (both v1 and v2 ImmGen releases). DICE reference log-transformed transcript-per-million data was downloaded from DICE database directly. Log-normalized counts for other references were obtained from SingleR package. Reference data frames were organized for CIPR pipeline using the code accessible at GitHub repository [19]. Abbreviations: Baso, basophil; Eosino, eosinophil; gd-T, gamma-delta T; Gran, granulocyte; ILC, innate lymphoid cells; Mac, macrophages; Mono, monocytes; NK, natural killer; Treg, regulatory T cells; Prog, progenitor; DC, dendritic cells; Neutro, neutrophil; H/M/E/SC, hematopoietic/mesenchymal/embryonic stem cells; MEP, megakaryocyte erythrocyte progenitor; CMP, common myeloid progenitor; GMP, granulocyte-macrophage progenitor; iPS, induced pluripotent stem cell. *Although the DICE reference data is originally composed of 1561 samples, to reduce compute time and generate readable outputs, we utilized mean transcript-per-million data per cell type resulting in 15 averaged samples).

### Use case scenario: melanoma tumor infiltrating lymphocyte scRNAseq data

**Fig. 1:**
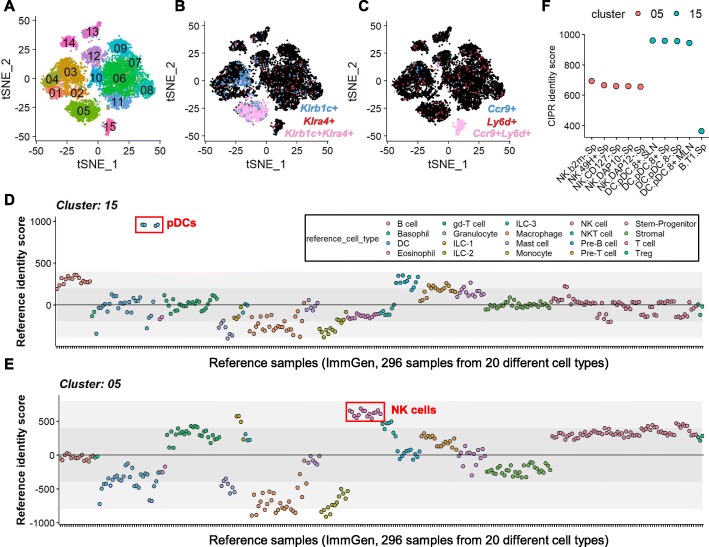
CIPR provides a R/Shiny-powered graphical user interface to facilitate cluster annotation in scRNAseq experiments. **a** T-distributed stochastic neighbor embedding (t-SNE) plot for the example scRNAseq data derived from murine melanoma tumor infiltrating lymphocytes shows 15 distinct immune cell clusters within the tumor microenvironment (the dataset contains 13,985 features and 11,054 cells) [28]. To demonstrate the capabilities of CIPR we focus on clusters 05 and 15 which distinctly expressed (**b**) natural killer cell (NK) and (**c**) plasmacytoid dendritic cell (pDC) markers respectively. **d** We used the CIPR pipeline to score the gene expression profiles of cluster 15 (pDC) against 296 mouse immune cells found in the ImmGen reference. CIPR algorithm calculates a distinct identity score for each reference cell type and generates a graphical summary of the results. In these plots, 4 highest data points (red rectangle) correspond to pDC samples within the ImmGen reference. The shaded regions in the graphs delineate 1 and 2 standard deviations around the mean identity score calculated from the entire reference data frame. Data points are color-coded based on the reference cell type allowing an easy assessment of the results. **e** The CIPR results for cluster 05 (NK cells) is shown. Marked data points depict the NK cells in the ImmGen dataset that had the highest identity scores. Users can visualize graphs for each cluster separately and have the option of further manipulating the plots if the R package implementation of CIPR is used. **f** CIPR can also generate graphical outputs to summarize the 5 top-scoring reference samples for each experimental cluster. The scatter plot shows the pDC and NK cell subsets that had the highest scores for clusters 05 and 15. In Shiny implementation of CIPR, users can draw rectangles around these points to prompt a table output which provides further information about the reference cell types on the graph

We have recently described immune cell dynamics during murine melanoma tumor growth in vivo [28]. In this study, CD45+ flow cytometry-sorted immune cells were sequenced via the 10X Genomics platform followed by computational analysis using Seurat R package [29]. Our analysis revealed 15 distinct single cell clusters within the tumor microenvironment **(**Fig. 1a**)**. To demonstrate the capabilities of CIPR, here we focus on clusters 05 and 15 which distinctly expressed the marker genes defining natural killer (NK) cell and plasmacytoid dendritic cell (pDC) lineages, respectively **(**Fig. 1b, c**)** [30, 31]. Using Seurat, we performed differential expression analyses at the cluster level and used this as the input for CIPR’s recommended logFC dot product method (see below for comparisons of different CIPR methods). In this analysis, we used Immunological Genome Project (ImmGen) reference which contains microarray data from sorted mouse immune cells (296 samples from 20 main cell types) [20]. CIPR calculates a distinct identity score for each unknown cluster-reference pair resulting in 296 calculations per cluster. The results for individual clusters are shown in scatter plots where data points correspond to the identity score calculated for a specific reference cell subset (plotted in the x-axis) **(**Fig. 1d, e**)**. The color-coded data points help users distinguish the enrichment of identity scores in particular reference cell types at a glance. To help assessing the quality of CIPR predictions, the algorithm outputs shaded regions in the scatter plots that mark the boundaries of 1 and 2 standard deviations of the identity score across the reference data frame. Furthermore, we implemented a z-score approach where the distance of the identity score for a particular reference cell type is calculated in standard deviation units from the average identity score across the whole reference dataset. As the identity- and z-score calculations are impacted by the composition of the input/reference data and the analysis parameters, it is difficult to define a widely applicable significance threshold for the predictions. In our hands, predictions with z-scores higher than 1 were consistent with expert knowledge-based manual annotations. In CIPR, we chose not to leave the low/intermediate-scoring clusters unlabeled since, even though the cluster in analysis may not have a perfect match in the reference subset, knowing which reference cell subset it resembles the most is informative, especially when performing iterative analyses. Indeed, recent studies show that analytical pipelines which implement an “unassigned” classification do not have an overall improvement in prediction accuracy [14, 16]. Thus, we anticipate that the graphical outputs of CIPR will provide a convenient and visual means to assess the strength of predictions in individual studies. In our experimental data, as expected, cluster 15 was strongly predicted to be a pDC subset as evidenced by the 4 blue-colored data points which were well above the rest of the reference subsets **(**Fig. 1d**)**. Cluster 05 scored the highest with the NK cell subsets which are depicted by pink-colored data points **(**Fig. 1e**)**. Although these scatter plots are informative, the user may only want to see the top scoring reference datasets for each cluster. CIPR pipeline also generates a summary output in which only the 5 highest-scoring reference cell types are plotted per cluster **(**Fig. 1f**)**. In the Shiny implementation of CIPR, users can draw a rectangle around these points which will prompt a table output below the figure providing further details about the reference cell types. The summary and per-cluster graphical outputs are created under distinct tabs in the CIPR-Shiny to speed up the user interaction and to create a minimalist interface. Users can choose to suppress one or both of these plots in the CIPR R package to tailor pipeline for programmatical use.

## Results and discussion

### Different computational approaches in CIPR

Selecting the genes or features that have higher discriminatory potential is a critical step in automated cluster classification algorithms. Some computational pipelines perform feature selection automatically, while others allow custom feature selection or proceed to analysis with no pre-filtering. Machine-learning based feature selection can improve algorithmic predictions, but it can also increase the demand for computing resources depending on the number of cells in the analysis, as reported by Zhao et al. [16] and Huang et al. [15]. In CIPR, we implemented two main computational approaches to calculate identity scores: i) analysis using data only from the differentially expressed genes in clusters, ii) analysis using the entire gene set (no feature selection). Our experience suggests that the genes with the least discriminatory power will be naturally filtered out in the first case, leaving the most informative genes that can better distinguish the cell clusters from one another in the analysis. The CIPR algorithm can compare the logFC values of differentially expressed genes in experimental clusters with the logFC values of the matching genes in the reference dataset (calculated by taking the ratio of gene expression in the reference subset to the average expression value across the entire reference data frame) by using one of three methods: i) dot product, ii) Spearman’s correlation, iii) Pearson’s correlation. We recommend using the logFC dot product method as it factors in both the direction and the amount of differential expression for a given gene. For instance, if a gene is highly upregulated or downregulated in the unknown cluster and in the specific reference sample, the multiplication of such logFC values will contribute to the overall identity score, while the genes showing a strong anti-correlation will proportionately reduce the identity score. CIPR can also test the linear and nonlinear relationships between the logFC values from the unknown cluster and the reference dataset using Spearman’s or Pearson’s respectively. Alternatively, CIPR pipeline can also assess the nonlinear and linear correlations between the input and reference datasets by considering all genes regardless of differential expression status.

**Fig. 2:**
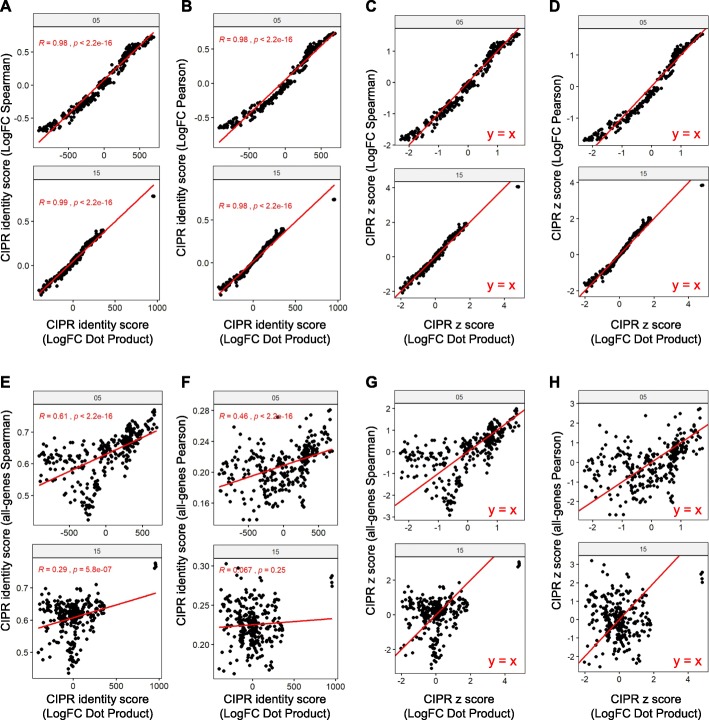
Different analytical methods implemented in CIPR performs comparably to annotate single cell clusters. Three of the analytical methods in CIPR (logFC dot product, logFC Spearman’s or Pearson’s correlation) utilizes only differentially expressed genes in clusters. The recommended approach in CIPR is logFC dot product method since it takes both the direction and the amount of differential expression into account when calculating identity scores per cluster. The other approaches in CIPR are designed to analyze the expression profiles of all the genes in the experimental data regardless of their differential expression status. This figure compares the predictions of the logFC dot product method to other analytical approaches in CIPR. Data points in the scatter plots indicate the identity score of individual ImmGen reference cell subsets calculated for clusters 05 and 15 by different methods. As expected, there is a strong correlation between the results of logFC dot product method and (**a**) logFC Spearman’s and (**b**) logFC Pearson’s correlation methods for both clusters. **c**, **d** The same strong correlation was observed when the z-scores were compared for these methods, although logFC dot product differentiated the highest scoring reference subsets slightly better as evidenced by a higher z-score. The results of (**e**) all-genes Spearman’s and (**f**) all-genes Pearson’s methods show an overall positive correlation with those from logFC dot product method, although logFC dot product approach was able to better differentiate the top-scoring reference subsets as evidenced by higher z-scores shown in panels **g** and **h**. Similar observations were made for other clusters in the experimental dataset but are not shown due to space constraints

We sought to determine how these different analytical approaches compare to the recommended logFC dot product method. As expected, all three logFC comparison methods showed a high degree of concordance for both clusters 05 (NK cells) and 15 (pDCs) **(**Fig. 2a, b**)**. When we examined the z-score distribution for these methods, we observed a similar trend with slightly higher z-scores for top hits when the logFC dot product method is used **(**Fig. 2c, d**)**. We then compared the logFC dot product method with the correlation methods that utilize the entire gene set (all-genes Spearman/Pearson methods). Although the CIPR identity scores between these approaches showed an overall positive correlation, this relationship was weaker at the low/intermediate-scoring reference subsets suggesting that logFC dot product method may have a higher discriminatory power compared to the all-genes correlation-based methods **(**Fig. 2e, f**)**. As expected, since the reference data set and the experimental data originated from different experimental approaches, Pearson’s correlation performed poorer compared to Spearman’s method **(**Fig. 2g, h**)**. Nevertheless, we anticipate that the all-genes Pearson’s method can be useful in some experimental contexts with custom-provided reference datasets. Similar trends were observed when other clusters in the scRNAseq dataset were examined (data not shown). These findings suggest that different computational approaches implemented in CIPR generates converging results and can be adapted to various experimental contexts.

### CIPR performs faster than other robust cluster annotation methods and produces comparable results

**Fig. 3:**
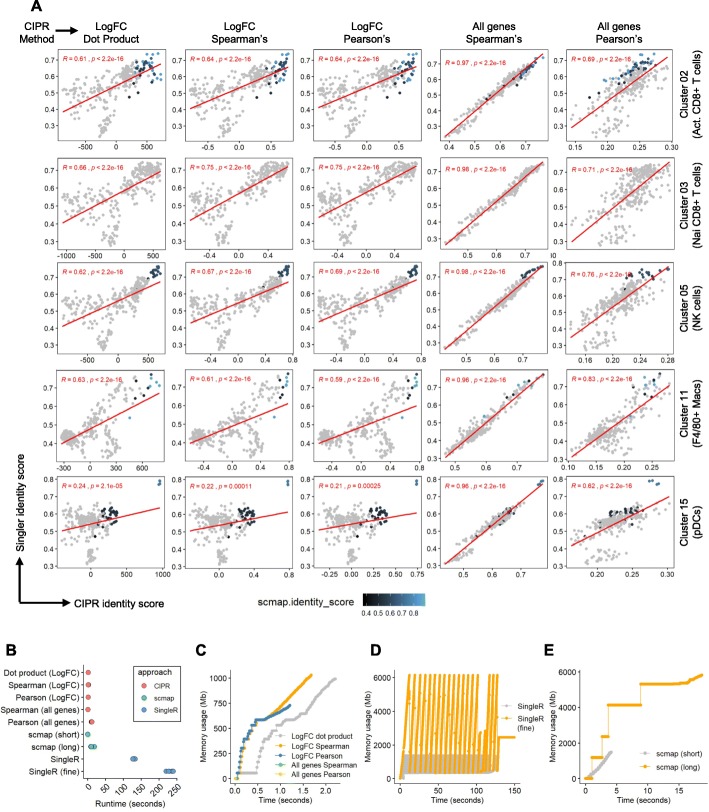
CIPR performs faster than other cluster analysis approaches and produces comparable results. **a** SingleR and scmap are recently described R packages for automated cluster analysis which can perform analyses at the cluster level similarly to the CIPR approach. These algorithms were shown to perform well in various experimental contexts and can serve as a high benchmark for automated cluster analysis solutions. By performing all the analyses at the cluster level, here we report a comparison of CIPR R package (v.0.1.0), SingleR (v1.0.5) and scmap (v1.8.0) in terms of predictions and performance. For these comparisons, a Surface Pro4 computer equipped with 64-bit Win7, 16 GB memory, 2.2GHz i7-6650U CPU, R (v.3.6.2), and RStudio (v.1.2.5033) was used with no other background processes. **a** Five analytical methods implemented in CIPR were compared to SingleR and scmap across 5 individual clusters. Data points indicate the identity scores calculated for each ImmGen reference cell subset by different methods. Color gradient specifies the identity score calculated by scmap method (gray indicates no significant mappings were found). As expected, CIPR’s all-gene Spearman’s/Pearson’s methods are highly concordant with SingleR pipeline. The results from CIPR logFC methods show an overall positive correlation with SingleR, where the highest scoring reference cell types in CIPR were similar to those calculated by SingleR and scmap. In some cases, scmap failed to find a significant association which may be due to its suboptimal power when a bulk reference data is used as input. **b** CIPR performs significantly faster than SingleR, and comparably to scmap in 5 separate tests. We benchmarked the runtime of SingleR function both with and without fine tuning feature. Scmap (short) measures the runtime of scmapcluster computational engine, whereas scmap (long) measures the runtime starting with the initial object creation. **c** CIPR utilizes less computer memory over time compared to (**d**) SingleR (no fine tuning) and (**e**) scmap

As reported by recent studies, automated cluster annotation algorithms SingleR [12] and scmap [13] perform robustly in various experimental contexts and simulations [14–16]. Scmap was initially developed for mapping different scRNAseq runs to one another, while SingleR allows using both bulk RNAseq and scRNAseq data as reference. SingleR calculates a Spearman’s correlation coefficient between single cell clusters and the reference samples after selecting variable features within the data frame [12]. Scmap method also performs unsupervised feature selection and scores the similarity of single cells to reference clusters by comparing its gene expression to the median expression within the reference dataset (13). As these solutions are conceptually similar to our approach and were shown to be accurate in their predictions, we next sought to determine how CIPR compares to these pipelines in terms of its predictions and performance. To be able to perform a fair comparison with CIPR, we adapted scmap pipeline to use ImmGen bulk microarray data as reference. The latest versions of CIPR (v0.1.0), SingleR (v.1.0.5) and scmap (v1.8.0) that were available for the most current stable release of R (v3.6.2) were used in benchmarking at the time of this writing. As the CIPR calculations are performed at the cluster level, we employed the same strategy for SingleR and scmap analyses. As expected, CIPR’s all-genes correlation method showed a strong concordance with SingleR method which also employs a correlation-based metric to calculate identity scores **(**Fig. 3a**)**. LogFC methods showed a significant overall positive correlation between CIPR and SingleR. The highest scoring reference samples were similar between all three methods in general. This was especially clear when analyzing the highly differentiated cell types such as NK cells (cluster 05), pDCs (cluster 15), and activated CD8+ T cells (cluster 02). Scmap did not find a significant association between naïve CD8+ T cell subset (cluster 03) and the reference dataset although naïve T cell subsets are present in the ImmGen reference data. However, the lack of a cell type assignment in this cluster and overall low scores observed using the scmap method, could be due to suboptimal feature selection when scmap is run with a bulk reference data *(personal communication, Dr. Martin Hemberg, the author of scmap package)*. The high concordance between CIPR and other established methods suggest that the CIPR algorithm provides accurate classifications across a variety of cell types.

When we compared the runtimes of these methods, we observed that CIPR was significantly faster compared to SingleR with or without SingleR’s “fine tuning” parameter **(**Fig. 3b**)**. Scmap had a similar speed with CIPR when only the identity prediction function is measured (labeled “scmap-short”) and had a slightly longer runtime when the time it takes to set up the analysis object is considered (labeled “scmap-long”, which would be a comparable use scenario to CIPR). However, we anticipate longer computing times from scmap pipeline when high dimensional scRNAseq data is used instead of the bulk reference data in current analyses (as per scmap design). We next assessed the memory utilization by these methods. All CIPR methods utilized less than 1000 megabytes (Mb) of memory **(**Fig. 3c**)**, whereas SingleR required ~ 1500 Mb without fine tuning and over 6000 Mb with fine tuning **(**Fig. 3d**)**. Scmap used comparable amounts of memory for computations but required 5000–6000 Mb of memory to set up the analysis object **(**Fig. 3e**)**. These observations suggest that the CIPR algorithm can perform cluster-level identity predictions in an accurate and efficient manner to facilitate scRNAseq data analysis. As the CIPR pipeline works with a simple R data frame structure, we anticipate that it will provide a quick and user-friendly solution for annotating cell clusters during iterative analyses.

### Filtering out genes with low variance can increase the discriminatory power of CIPR

**Fig. 4:**
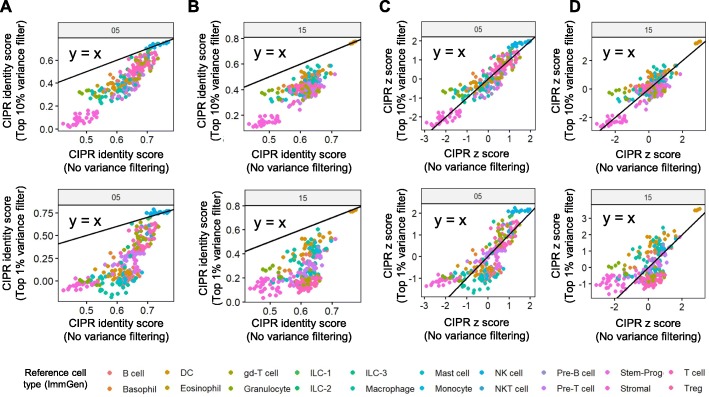
CIPR allows users to limit the analysis to highly variable reference genes to improve cluster annotations. As genes with variable expression profiles contain more information to discriminate cell types, we implemented a variance filtering parameter in CIPR. The user-defined variance threshold parameter instructs the algorithm to utilize the genes with variances above a certain quantile across the reference dataset, thus limiting the analysis to highly variable genes. Plots compare the CIPR results with or without variance thresholding when the all-genes Spearman’s method is used. Identity- and z-scores were calculated for clusters 05 (NK cells) and 15 (pDCs) using ImmGen reference and results for individual reference samples types are plotted as color-coded data points. Applying variance thresholding and increasing its stringency from top 10% to top 1% reduced the identity scores of low/intermediate-scoring reference cell subsets while the highest scoring reference cell subsets remained unaffected as evidenced by data points overlapping with y = x line for (**a**) cluster 05, and (**b**) cluster 15. Similar trends were observed for other clusters in analysis (not shown). The differential impact on identity scores of high- and low-scoring reference cell subsets lead to an increased z-score for the highest-scoring reference subsets for both (**c**) cluster 05 and (**d**) cluster 15. These findings suggest that variance thresholding can improve the discrimination of some reference cell subsets. Although the best thresholding value remains to be determined in individual studies, CIPR pipeline allows a level of flexibility to be adapted to different experimental contexts

In addition to limiting the CIPR pipeline to differentially expressed genes in the experimental data via logFC methods, users can choose to apply gene filtering based on the expression variance across the reference dataset. By eliminating the lowly variable genes in the reference, hence the noise, we hypothesize that all-genes correlation methods could perform better. We designed a numeric slider input in the Shiny applet (and a numeric input argument in CIPR R package) to allow the user to define a variance cutoff to keep top n^th^ percent of highly variable genes in the analysis. To test the effects of reference gene filtering on the CIPR results, we compared CIPR identity scores and z-scores calculated by the all-genes Spearman’s correlation method without variance filtering and with variance cutoff set to top 10% or 1% (leaving 2420 and 242 genes of the ImmGen reference in analysis respectively), and re-analyzed the clusters 05 (NK cells) and 15 (pDCs) introduced in Fig. 1. With the increasing stringency of variance filtering, the identity scores of the highest-scoring reference cell subsets remained unchanged whereas the identity scores of the intermediate-scoring reference cell subsets were reduced **(**Fig. 4a, b**)**. The z-scores of the highest scoring reference cell types were also found to generally increase with variance filtering as observed in both clusters 05 and 15 **(**Fig. 4c, d**)**. These findings suggest that reference feature subsetting based on expression variance can improve the discrimination of some cell clusters in scRNAseq data.

### Excluding irrelevant reference cell subsets may improve the CIPR predictions

One of the challenges in scRNAseq data analysis is finding a suitable reference data frame for experimental data at hand. Having irrelevant cell types in the reference dataset can unnecessarily increase the time and the computational resources necessary for the analysis, and can impact the classification quality [15, 16]. Therefore, focusing the analysis on a smaller subset of the reference dataset can be desirable in certain contexts. We implemented a user-friendly approach to limit the CIPR pipeline to the reference samples of interest via a drop-down menu in the Shiny implementation, and via a dedicated function argument that accepts human-readable strings in the R package implementation. Since the pairwise cluster-vs-reference correlations will be the same regardless of the composition of the reference dataset, CIPR’s all-genes based correlation methods are not expected to change upon reference subsetting. However, when using the logFC comparison methods, CIPR calculates differential gene expression per reference sample by comparing the gene expression values to the average gene expression across the reference dataset in analysis. Therefore, changing the composition of the reference dataset will impact the calculations of the logFC-based methods. We hypothesize that such reference subsetting can better discriminate closely related cell types, especially when analyzing datasets with low heterogeneity.

**Fig. 5:**
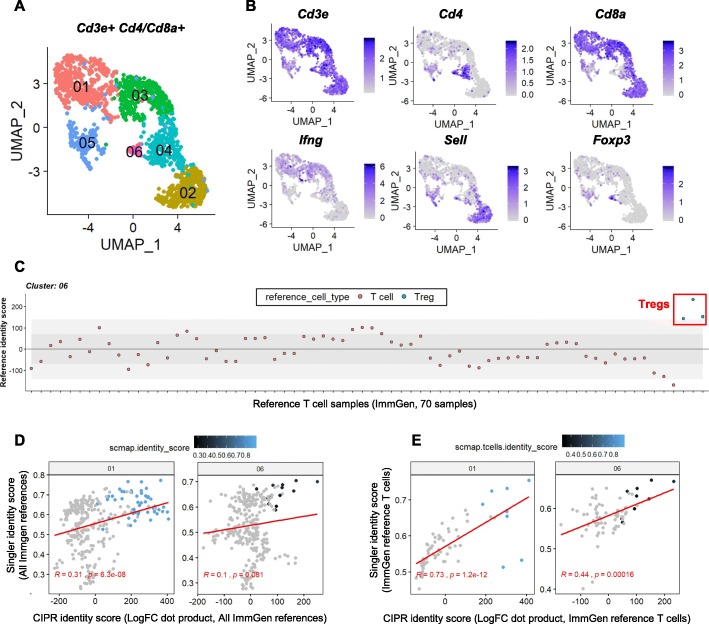
Irrelevant reference subsets can be excluded to tailor CIPR pipeline to different user needs. CIPR pipeline allows users to easily exclude the reference subsets that are of no interest for the study at hand. Limiting the analysis only to the relevant reference subsets can increase the readability of the graphical outputs and may better differentiate closely related single cell clusters. To demonstrate this capability, we subsetted the scRNAseq dataset described in Fig. 1 to contain only T cells (as defined by the simultaneous expression of *Cd3e* and *Cd4* or *Cd8a* marker genes). We then performed CIPR analyses with or without limiting the pipeline to T cell references within the ImmGen dataset. **a** Uniform manifold approximation and projection (UMAP) plot with 6 distinct single-cell clusters shows the heterogeneity within the T cell subsets in the tumor microenvironment. **b** Representative feature plots indicate that the clusters are composed of *Cd4*+ helper and *Cd8a*+ cytotoxic T cells some of which exhibited an activated phenotype (*Ifng*+ cells) while others appeared to have naïve-memory phenotype (*Sell*+ cells). Of note, cluster 06 is composed of *Foxp3*+ regulatory T cells (Tregs). **c** CIPR analysis using logFC dot product method shows that highest scoring reference subsets for cluster 06 are regulatory T cell subsets within the ImmGen reference data. **d** Graphs show that identity scores calculated by CIPR, SingleR and scmap are positively correlated for both cluster 01 (activated *Cd8a*+ cells) and cluster 06 (Tregs). For these analyses, the entire ImmGen reference data (296 samples spanning 20 different cell types) were used, and the calculations were performed at the cluster level as described above. **e** The positive correlation between different analytical approaches were stronger when the reference dataset was limited to T cell subsets (70 samples in ImmGen data). In general, the highest scoring reference cell subsets in CIPR also scored the highest in scmap and SingleR methods

To test the performance of CIPR with a limited set of reference samples, we subsetted all T cells from the tumor-infiltrating immune cell scRNAseq data described in Fig. 1 [28], as identified by the concomitant expression of *Cd3e* and *Cd4* or *Cd8a* marker genes **(**Fig. 5a**)**. We performed new dimensionality reduction and clustering analyses and examined known marker gene expression which revealed that the dataset contains various *Cd4* and *Cd8a*-expressing T cell subsets including L-selectin-high (*Sell*, Cd62l) naïve T cells (clusters 01 and 03), interferon gamma (*Ifng*)-high activated T cells, and *Foxp3*-high regulatory T cells (clusters 05 and 06) **(**Fig. 5b**)**. When we performed CIPR analysis using 70 T cell reference samples found in ImmGen reference, we observed that regulatory T cells (Tregs) scored the highest in cluster 06 as expected **(**Fig. 5c**)**. Using T cell-specific scRNAseq data, we then compared the performance of CIPR, SingleR, and scmap with unfiltered ImmGen reference (296 samples). Although the highest scoring reference cell types were similar among the different methods, the positive correlation between CIPR logFC dot product method and SingleR were weaker compared to the parental experimental dataset with high heterogeneity **(**Fig. 5d**)**. When we performed the analyses after limiting the reference dataset to T cells for all the methods in comparison, the concordance between CIPR and SingleR increased for both activated T cells (cluster 01) and Tregs (cluster 06) **(**Fig. 5e**)**. For these analysis we excluded T cell-like subsets that exhibit a transitionary phenotype (such as gamma delta T cells and natural killer T cells), as these cells do not typically express the T cell co-receptor genes (*Cd4* and *Cd8a*) used for subsetting the T cells in our experimental data. Although further characterization is needed to elucidate how well the reference filtering approach can discriminate highly similar cell subsets, these findings suggest that CIPR can be adapted to various experimental contexts by the end user.

## Conclusions

CIPR is a web-based Shiny applet/R package that can be used to quickly and accurately annotate unknown single cell clusters in scRNAseq experiments without prior knowledge of biological markers for the investigated cell types. CIPR provides a user-friendly graphical interface to score cluster-specific gene expression patterns against known reference cell subsets and generate informative outputs. Quality control metrics and graphical outputs implemented in CIPR help assess the confidence of the predictions in individual studies. User-defined gene/reference subsetting functionality allows adapting the CIPR pipeline to various experimental contexts. Benchmarking CIPR against other robust software solutions that perform a similar task suggests that our pipeline generates comparable results in a significantly shorter timeframe and requires considerably less computational resources. Thus, CIPR is ideal for iterative analyses where the user wants to test different clustering parameters and quickly assess the identity of calculated cell clusters. We provide detailed vignettes to prepare CIPR-ready simple data frames on the Shiny web platform and within the CIPR package which do not require any more programming skills than what is needed to run other tools. Furthermore, the R package implementation of CIPR enables users to easily integrate our algorithm into existing analytical pipelines without leaving the R environment and allows flexible graphing options. In summary, CIPR can facilitate scRNAseq data analysis by quickly and objectively annotating single cell clusters.

## Availability and requirements

**Project Name:** Cluster Identity Predictor (CIPR).

**Project Home Page:** https://aekiz.shinyapps.io/CIPR/

**Project Repository (Shiny app):** https://github.com/atakanekiz/CIPR-Shiny

**Project Repository (R package):** https://github.com/atakanekiz/CIPR-Package

**Operating Systems:** Platform independent (web-based).

**Programming Language:** R.

**Other requirements:** Browser, internet access. If running the R code locally though CIPR R package, package dependencies such as dplyr, tibble, ggpubr, and gtools.

**License:** GNU GPL.

**Restrictions for non-academics:** None.

## Data Availability

Reference datasets utilized in CIPR are publicly available [20–27]. ScRNAseq data derived from tumor infiltrating immune cells were recently published [28] and accessible at gene expression omnibus (GEO) database (GSE121478) [32]. To provide example data for CIPR and to reduce compute times, this experimental dataset was trimmed to contain only 4 single cell clusters in shiny implementation of CIPR, and is available at CIPR GitHub repository [19]

## Abbreviations

ScRNAseq: Single cell RNA sequencing
CIPR: Cluster Identity Predictor
ImmGen: Immunological Genome Project
logFC: Log fold-change
NK cells: Natural killer cells
pDC: Plasmacytoid dendritic cells
DICE: Database of Immune Cell Expression
PBMC: Peripheral blood mononuclear cells
ENCODE: The Encyclopedia of DNA Elements
